# Supplementary material for: Microbial Colorants Production in Stirred-Tank Bioreactor and Their Incorporation in an Alternative Food Packaging Biomaterial
Source: J Fungi (Basel). 2020 Nov 2;6(4):264. doi: 10.3390/jof6040264 (PMC7712370; doi:10.3390/jof6040264)
Supplement: Supplementary file 1 [file jof-06-00264-s001.pdf]

Journal of *Fungi*

MDPI

Table S1. Consumption of sucrose and pH over time (0 to 240 h) of submerged culture of *T. amestolkiae* in stirred-tank bioreactor varying the stirring speed (100 to 600 rpm) at 2.0 vvm and 30°C.

Sucrose (g/L)											
**Stirring speed (rpm)**	**Time (h)**										
	**0**	**24**	**48**	**72**	**96**	**120**	**144**	**168**	**192**	**216**	**240**
100	48.58±1.41^{a}	39.46±1.98^{a}	29.05±0.98^{a}	25.29±0.56^{a}	11.80±0.32^{a}	7.32±0.14^{a}	0.00±0.00^{a}	0.00±0.00^{a}	0.00±0.00^{a}	0.00±0.0^{0a}	0.00±0.00^{a}
200	48.44±1.20 ^{a}	44.31±1.37^{b}	30.53±0.76^{a,b}	24.80±0.55^{a}	12.21±0.22^{a}	7.15±0.06^{a}	6.16±0.14^{b}	3.44±0.04^{b}	3.18±0.12^{b}	2.53±0.29^{b}	2.64±0.13^{b}
300	48.44±1.20 ^{a}	43.84±1.81^{b}	27.20±2.02^{a,c}	19.55±1.90^{b}	5.35±0.44^{b}	4.90±0.21^{b}	3.94±1.14^{c}	1.22±0.27^{c}	1.69±0.16^{c}	1.45±0.19^{c}	1.56±0.07^{c}
400	48.43±1.20 ^{a}	37.67±3.96^{a}	25.21±0.69^{c}	12.34±1.25^{c}	7.84±1.78^{c}	3.71±0.07^{c}	3.56±0.34^{c}	3.25±0.49^{b}	2.82±0.13^{d}	2.29±0.10^{b}	2.24±0.06^{d}
500	48.84±0.98 ^{a}	37.67±2.96^{a}	29.84±0.05^{a}	19.06±0.09^{b}	8.17±0.63^{c}	3.70±0.09^{c}	2.88±0.01^{c}	2.38±0.05^{c,d}	2.17±0.16^{e}	1.87±0.01^{c}	1.82±0.00^{e}
600	48.19±0.94 ^{a}	37.01±0.14^{c}	22.02±0.38^{d}	14.60±0.04^{d}	5.54±0.27^{b}	2.31±0.36^{d}	2.02±0.23^{d}	2.01±0.45^{d}	1.96±0.37^{c,e}	1.38±0.15^{c}	0.89±0.23^{f}

pH											
**Stirring speed (rpm)**	**Time (h)**										
	**0**	**24**	**48**	**72**	**96**	**120**	**144**	**168**	**192**	**216**	**240**
**100**	4.50±0.00 ^{a}	4.57±0.30^{a}	5.46±0.64^{a}	5.80±0.67^{a}	5.98±0.31^{a}	6.00±0.27^{a}	6.01±0.27^{a}	6.15±0.33^{a}	6.07±0.52^{a}	6.76±0.80^{a}	6.76±0.83^{a}
**200**	4.50±0.00 ^{a}	4.67±0.19^{a}	5.40±0.52^{a}	5.79±0.57^{a}	5.99±0.13^{a}	6.02±0.19^{a}	6.01±0.10^{a}	6.12±0.28^{a}	6.02±0.44^{a}	6.69±0.30^{a,b}	6.71±0.69^{a}
**300**	4.50±0.00 ^{a}	4.50±0.03^{a}	5.57±0.29^{a}	5.97±0.14^{a}	6.07±0.14^{a}	5.9±0.25^{a}	5.97±0.14^{a}	6.06±0.29^{a}	5.97±0.14^{a}	6.96±0.13^{a}	6.97±0.14^{a,b}
**400**	4.50±0.00 ^{a}	4.71±0.01^{a}	5.19±0.01^{a}	5.50±0.03^{a}	5.85±0.01^{a}	6.02±0.06^{a}	6.11±0.01^{a}	6.24±0.04^{a}	6.30±0.01^{a,b}	6.38±0.01^{a,b}	6.41±0.04^{a}
**500**	4.50±0.00 ^{a}	5.25±0.09^{b}	5.65±0.04^{a}	5.74±0.03^{a}	5.81±0.03^{a}	5.90±0.01^{a}	5.76±0.12^{a}	6.12±0.01^{a}	6.91±0.01^{a}	7.47±0.01^{a}	7.80±0.01^{c}
**600**	4.50±0.00 ^{a}	4.82±0.06^{a}	5.00±0.04^{a}	5.45±0.14^{a}	7.25±0.05^{b}	7.60±0.04^{b}	8.14±0.12^{b}	8.20±0.04^{b}	8.32±0.04^{c}	8.32±0.05^{c}	8.30±0.05^{d}

Statistical test Anova one way (with Welch correction for unequal variances). The results are presented by means and standard deviations of triplicates. Equal letters in the same column represent values equal to a significance level of 5%.

Journal of *Fungi*

Table S2. Peroxide content in the butter as a function of the packaging film used, such as: cassava starch-based films with colorant additives (CFC), cassava starch-based films without colorant additives (CF), conventional plastic (CP), and unpackaged butter (U), during different periods of time (0, 7, 15, 30, and 45 days) in storage.

Films	Day 0	Day 7	Day 15	Day 30	Day 45
Formulation	Peroxide content (meq/kg)				
CFC	0.627 ± 0.018^{a}	0.725 ± 0.029^{a}	0.796 ± 0.070^{a}	0.886 ± 0.044^{a}	1.370 ± 0.004^{a}
CF	0.627 ± 0.018^{a}	$0.772 \pm 0.026^{a,b}$	0.983 ± 0.057^{b}	0.951 ± 0.010^{b}	2.076 ± 0.074^{b}
CP	0.627 ± 0.018^{a}	0.812 ± 0.016^{b}	1.036 ± 0.071^{b}	1.121 ± 0.021^{c}	2.130 ± 0.149^{b}
U	0.627 ± 0.018^{a}	0.848 ± 0.010^{c}	1.008 ± 0.087^{b}	1.022 ± 0.011^{c}	2.692 ± 0.122^{c}

Statistical test Anova one way (with Welch correction for unequal variances). The results are presented by means and standard deviations of triplicates. Equal letters in the same column represent values equal to a significance level of 5%.

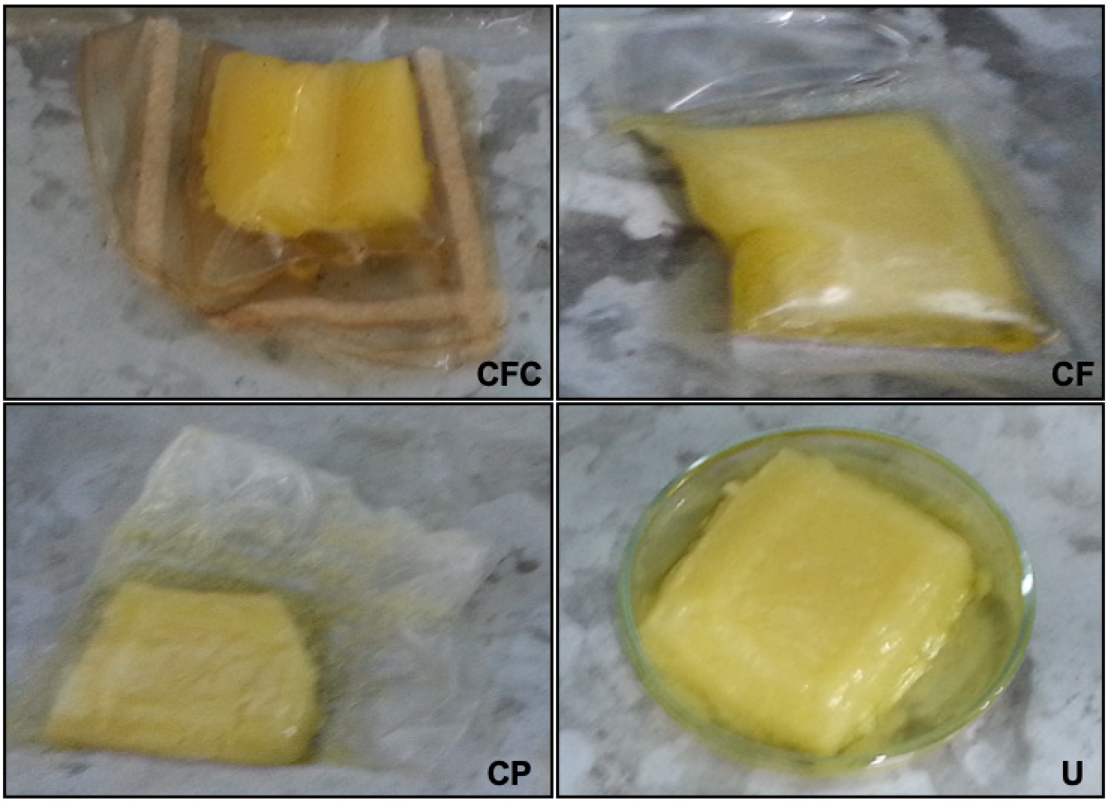

Figure S1. Butter packaged in cassava starch-based films with colorant additives (CFC), cassava starch-based films without colorant additives (CF), conventional plastic (CP), and unpackaged butter (U).

© 2020 by the authors. Submitted for possible open access publication under the terms and conditions of the Creative Commons Attribution (CC BY) license (http://creativecommons.org/licenses/by/4.0/).
